# Technology-Assisted Self-Monitoring of Lifestyle Behaviors and Health Indicators in Diabetes: Qualitative Study

**DOI:** 10.2196/21183

**Published:** 2020-08-28

**Authors:** Yan Du, Brittany Dennis, Shanae Lakel Rhodes, Michelle Sia, Jisook Ko, Rozmin Jiwani, Jing Wang

**Affiliations:** 1 Center on Smart and Connected Health Technologies School of Nursing University of Texas Health Science Center at San Antonio San Antonio, TX United States; 2 School of Nursing University of Texas Health Science Center at San Antonio San Antonio, TX United States

**Keywords:** technology, monitoring, lifestyle, diet, exercise, weight, glucose, diabetes management

## Abstract

**Background:**

Self-monitoring is key to successful behavior change in diabetes and obesity, and the use of traditional paper-based methods of self-monitoring may be time-consuming and burdensome.

**Objective:**

This study aimed to explore participant experiences while using technology-assisted self-monitoring of lifestyle behaviors and health indicators among overweight or obese adults with type 2 diabetes.

**Methods:**

Qualitative data collected from the intervention group of a 6-month, three-arm (control, paper diary, and technology-assisted self-monitoring groups) randomized clinical trial were analyzed. Study participants in the intervention group monitored their diet, exercise, and weight using the LoseIt! app, and their blood glucose levels using a glucometer and the Diabetes Connect app. Semistructured group discussions were conducted at 6 weeks (n=10) from the initiation of the behavioral lifestyle intervention and again at 6 months (n=9). All group interviews were audiotaped and transcribed verbatim. Using a combination of thematic and comparative analysis approaches, two trained professionals coded the transcriptions independently and then discussed and concluded common themes for the 6-week and 6-month discussions separately.

**Results:**

The sample (n=10), which primarily involved African American participants (n=7) and female participants (n=8), had a mean age of 59.4 years. The following eight themes emerged: (1) perceived benefits of technology-assisted self-monitoring; (2) perceived ease of use (eg, barriers: technical difficulties and lack of self-discipline; facilitators: help from family, friends, and the program); (3) use of technology-assisted self-monitoring; (4) facilitators of engaging in healthy lifestyle behaviors (eg, visualization and awareness of calorie input/expenditure); (5) positive lifestyle change; (6) barriers of engaging in healthy lifestyle behaviors (eg, event influence); (7) learning curve; and (8) monitored data sharing. The first six of these themes were shared between the 6-week and 6-month timepoints, but the codes within these themes were not all the same and differed slightly between the two timepoints. These differences provide insights into the evolution of participant thoughts and perceptions on using technology for self-monitoring and subsequent behavioral lifestyle changes while participating in lifestyle interventions. The findings from the 6-week and 6-month data helped to paint a picture of participant comfort and the integration of technology and knowledge overtime, and clarified participant attitudes, difficulties, behavioral processes, and modifications, as well as health indicators that were experienced throughout the study.

**Conclusions:**

Although there were some barriers, participants were able to identify various individual and external facilitators to adjust to and engage in technology-assisted self-monitoring, and it was concluded that the technology-assisted self-monitoring approach was beneficial, safe, and feasible to use for positive lifestyle change. These patient perspectives need to be considered in future research studies when investigating the effectiveness of using technology-assisted self-monitoring, as well as in clinical practice when recommending technology-assisted self-monitoring of lifestyle behaviors and health indicators to improve health outcomes.

## Introduction

Diabetes has become a worldwide public health concern, contributing to 10% of global health expenditure [1]. Approximately 31 million adults are living with diabetes in the United States, with an additional 88 million adults living with prediabetes, and their numbers are expected to increase greatly in the future [1,2]. The burden of diabetes is high and can be attributed to underlying complications and exacerbation of coexisting conditions. The total direct and indirect costs of diagnosed diabetes nationally increased from US $261 billion in 2012 to US $327 billion in 2017 [2].

Type 2 diabetes accounts for over 90% of all diabetes cases [2], and mounting evidence shows that most risk factors for type 2 diabetes are modifiable [3-5]. Some common modifiable risk factors for diabetes-related complications are being overweight or obese (BMI of 25.0 kg/m^2^ or over), having an unhealthy diet, being physical inactivity, and having a glycated hemoglobin (HbA_1c_) value of 7.0% or higher [2]. Research has demonstrated the success of self-monitoring interventions in influencing modifiable behavior change, weight management, and HbA_1c_ control in diabetes [6-10].

Self-monitoring approaches for lifestyle behaviors (eg, diet and physical activity), body weight, and blood glucose have been identified as some of the strongest predictors of weight loss and HbA_1c_ management [8,11]. For instance, a systematic review evaluating the effectiveness of self-monitoring interventions demonstrated a decrease in total sedentary time in the intervention group compared with the finding in the control group [7]. Consequently, behavioral modifications can lead to improved diabetes health outcomes, including but not limited to body weight, glycemic control, and prevention of diabetes-related complications [11-14]. Self-monitoring of blood glucose can lead to weight loss and better HbA_1c_ levels through increased adherence to dietary recommendations [8,10,12,15]. Furthermore, evidence suggests that the use of interventions involving self-monitoring of blood glucose leads to decreased rates of morbidity, mortality, and diabetes-related complications [12,13,16].

Despite the benefits of traditional paper-based methods of self-monitoring on healthy lifestyle behaviors [17-19], recent studies have revealed weaknesses and limitations in utilizing paper-based methods, such as untimeliness, time consumption, falsification of frequency and time, and lack of veracity [19,20]. On the contrary, compared with conventional approaches (eg, paper-based approaches) of health and behavioral management, technology-based methods have been drawing increasing attention owing to rapidly evolving innovation in the technological advances of self-monitoring. Studies have identified numerous benefits in both type 2 diabetes and weight control when utilizing technology for self-monitoring [8-10]. Accessibility and portability are the key features of technology-based methods when addressing issues encountered with paper-based methods. Technology-based self-monitoring is also more objective, offering customizable options for the user [6,21-23]. Users are able to set goals, view and sync real-time data for analysis and comparison, and engage in immediate reinforcement of healthy behaviors [9,22,24]. In addition, the burdens of locating appropriate references and performing calculations are conveniently accessible and automated through software applications, and they are compatible for use on multiple electronic devices [6,24,25].

Among the numerous advantages, some disadvantages of utilizing technology in self-monitoring were also revealed and were typically categorized as individual-specific or product-specific barriers. Individual-specific barriers include failure to record accurate or all data, decreased use over time, perceptions of the disease (not needing to self-monitor), skepticism of technology, and lack of technology or health literacy [25,26]. Discontentment with devices was also identified as a common barrier [26]. However, other studies contradict this finding of individual-specific barriers and suggest that more users are satisfied with the esthetics of how data are presented (eg, visual displays and graphs), reporting greater gratification of self-monitoring apps, especially when they are recommended by providers [25,26]. Product-specific barriers include users needing to constantly wear or carry devices for data processing, inaccuracy of the data captured, and data connectivity issues for specific geographical populations [24,25]. According to the European Association for the study of Diabetes and the American Diabetes Association, major barriers of concern include potential security breaches, inadequate processes of standardization, and exclusion of evidence-based practices; however, feasible recommendations have been provided to rectify these issues [21]. Some research has considered the difficulty in the use of technology as an age-related barrier, specifically for engaging in technology-based self-monitoring [7], and some studies have reported other barriers such as trial-and-error frustration levels and lack of knowledge, which can be potentially overcome by clear instructions and repetition [27,28]. A recent study evaluating mobile use and synchronization of virtual tools in a primarily older underserved population of adults who had comorbid overweight or obesity with type 2 diabetes reported high retention rates (96% at 3 months and 92% at 6 months) regarding patient engagement when using mobile technology [29].

The above advantages and disadvantages of using technology in self-monitoring are consistent with the elements in the technology acceptance model (TAM). This model includes five major related components as follows [30-32]: A person’s *intent to use* (acceptance of technology) predicts the *usage behavior* (actual use) of a technology, which is driven by a person’s perceptions of the specific technology’s *ease of use* and *usefulness* (benefits from using the technology), and lastly, the perceptions of ease of use and usefulness are affected by *external variables* such as individual differences (eg, age, gender, and education). The TAM, an information technology framework created to understand how users accept and use technology, has been widely utilized as a way to assess health technology usage, especially in the rapid evolution of health technology within the health care system [25,31-34].

The rapid evolution of technology and increasing dependence on smart devices continue to create a pathway for new developments and exploration in health care advancements. However, despite documented findings of the benefits and barriers of using self-monitoring through technology, there are gaps regarding the learning process of using technology-assisted self-monitoring of lifestyle behaviors and health indicators, and the potential of incorporating tracked and recorded data into health care. Therefore, this study aimed to explore participant experiences of using technology-assisted self-monitoring of lifestyle behaviors and health indicators among overweight or obese adults with type 2 diabetes at 6-week and 6-month timepoint discussions during a lifestyle intervention.

## Methods

### Study Population

Participants were recruited from an American Diabetes Association-certified diabetes education program in a community health center primarily serving uninsured or underinsured individuals living in Harris County, Texas. A total of 26 participants were recruited and randomized to a control group (n=6), a paper diary group (n=9), or an intervention group (n=11; one withdrew). Participants in the intervention group were instructed to use a smartphone for self-monitoring of diet, exercise, and weight through the LoseIt! app (FitNow, Inc). Participants were also given a Bluetooth-enabled glucometer (Entra Health Systems LLC) to self-monitor blood glucose. The device transferred glucose data to the Diabetes Connect app (PHRQL Inc) automatically with the touch of a button. Table 1 illustrates the functions, features, and participant responsibilities for each of the devices and apps used in the study. The details of the study design and intervention have been reported previously [29]. Consent was obtained from each participant, and the study was approved by the Institutional Review Board of the University of Texas Health Science Center at Houston.

**Table 1:** Summary of the features, functions, and participant responsibilities for devices and apps.

Device or app	Features and functions	Participant responsibilities
LoseIt! app	Monitoring of diet, exercise, and weight in one app	Diet: log all food intake Exercise: log exercise type and duration Weight: enter weight scale reading in the app
Bluetooth-enabled glucometer	Finger stick-based glucometer Bluetooth function	Test blood glucose; testing frequency is recommended by the primary care physician (minimum once daily). Open Diabetes Connect app and Bluetooth on the smartphone. After testing, hit a button on the glucometer so that data are automatically transferred from the glucometer to the Diabetes Connect app
Diabetes Connect app	Receives and stores glucose information	Use the app to track blood glucose values.
Weight scale	Regular weight scale	Encouraged to use the scale daily to take weight measurement and manually enter values in the LoseIt! app.

### Data Collection

Qualitative data were collected between January 2013 and August 2013 from the intervention group (technology-assisted self-monitoring) at the following two timepoints: 6 weeks and 6 months after initiation of the lifestyle intervention. The study principal investigator facilitated focus group discussions using a semistructured interview guide. First, interviews were conducted with the 10 intervention participants 6 weeks after beginning the intervention, during which questions on six topics, including experience using the health devices, factors affecting monitoring and recording of weight, and experience of self-monitoring blood glucose, were covered in the group discussion. Questions such as “What was your experience using the smart phone?” were asked, and follow-up probe questions were used whenever appropriate. Second, 6 months after initiation of the intervention, participants were invited to another focus group discussion again involving a semistructured interview. Nine participants attended the 6-month focus group discussion (n=9), and one make-up individual interview (n=1) was conducted. In addition, participant preference of sharing tracked health information was explored at 6 months by asking questions like “Who would you like to share this information with?” in regard to participant health data. The interview time for group discussions was approximately 45 minutes, and the one make-up individual interview was about 10 minutes. Similar interview question guides were used during both interviews. The question guide topics are summarized in Textbox 1. All group and make-up discussions were audiotaped and transcribed verbatim in Microsoft Office 365 Word Version 1902 (Microsoft Corporation) for analysis.

**Question guide topics for focus group discussions and one make-up individual interview.**

**Question topics**

- Experience of using a phone or the LoseIt! app
- Experience of monitoring and recording weight
- Experience of self-monitoring blood glucose, and use of the Diabetes Connect app with a glucometer
- Factors affecting engaging in monitoring and recording
- Feedback regarding group sessions (only for 6 weeks)
- Comparison regarding individual versus group sessions
- Safety and security of personal health information (only for 6 months)
- Voluntary sharing of personal health information (only for 6 months)

### Data Analysis

A combination of inductive and deductive thematic analyses along with a constant comparative analysis approach was used to analyze the data, incorporating both the data-driven inductive method and the deductive a priori template of codes [35,36]. Data analysis was conducted separately for the 6-week and 6-month data. The same step-by-step analysis procedures were used for analyzing each data set, and they are described below.

First, a graduate research assistant with prior qualitative analysis experience and a junior faculty member with years of qualitative study experience coded the data independently using an open coding method. Discrepancies were discussed and an agreement for each discrepancy was reached. Different and similar codes between the two timepoints were compared and discussed. Consultation with a senior qualitative scientist was initiated as deemed necessary. An initial code book was created for both 6-week and 6-month timepoints. Thereafter, codes were reconciled between the researchers and further grouped into higher order headings according to the TAM. Given that this study attempted to explore participant experiences of using self-monitoring of multiple healthy behaviors and health indicators, the TAM was not able to capture all emerged codes. Therefore, the research team modified the TAM based on the initial codes in this study.

Second, the modified TAM was further used by the two researchers to guide the second round of coding, but this time to capture some specific information in the modified model, which might not have been captured during the initial coding. The principle was not to force any concept to fall into the model. Codes, categories, and themes that emerged within each of the two data sets were further discussed between the two coders. Differences and similarities between the two data sets were also discussed and compared. A senior scientist was consulted and data were referred to whenever necessary during the analysis process. Agreement was achieved for all themes, categories, and codes within both the 6-week and 6-month discussions.

## Results

### Sample

The sample (n=10), which primarily included African American participants (n=7) and female participants (n=8), had a mean age of 59.4 years and average BMI of 37.9 kg/m^2^. Participant adherence to technology-assisted self-monitoring has been reported previously [29]. The median percentages of days with at least one self-monitoring entry for diet, physical activity, weight, and glucose were 96.6%, 37.3%, 49.7%, and 72.7%, respectively [29]. 

### Themes

**Figure 1:**
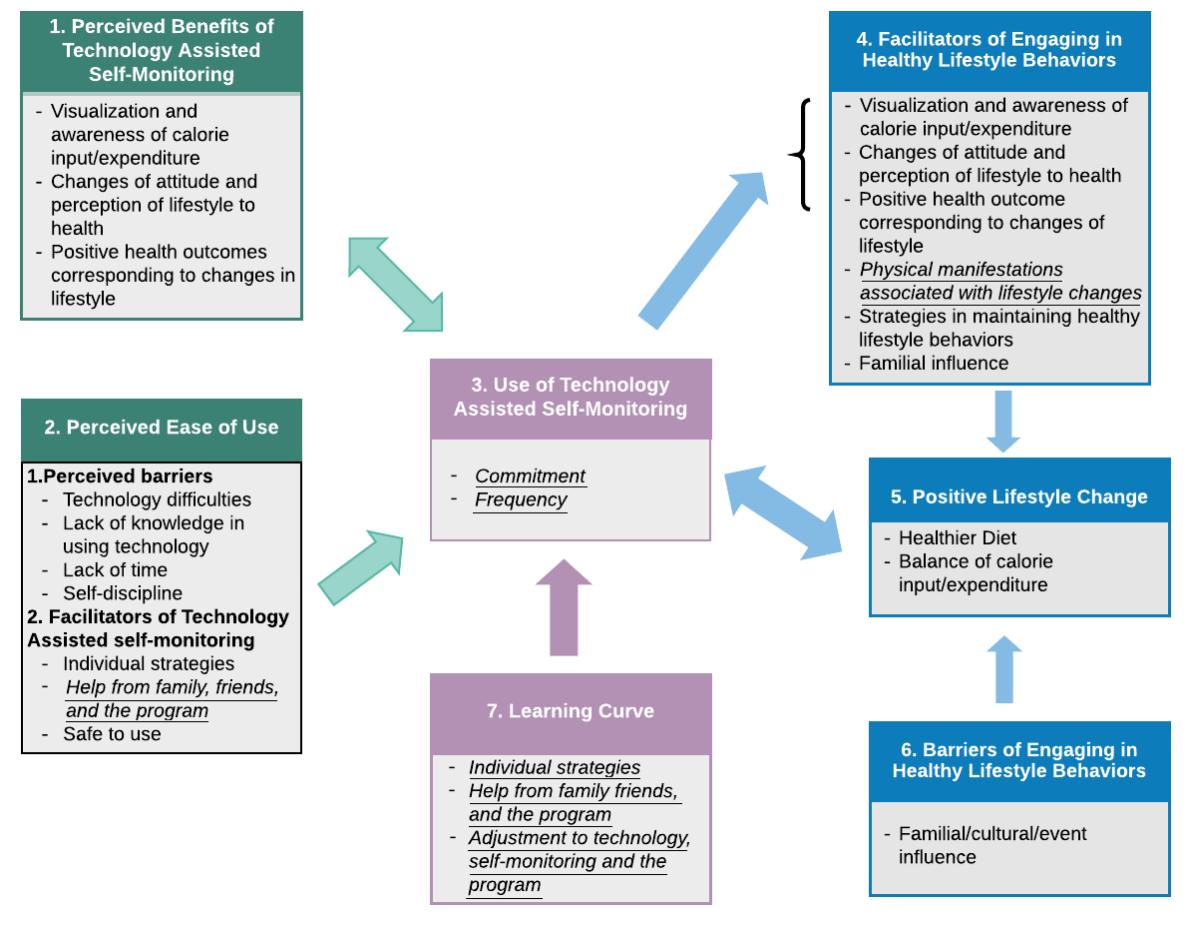
Themes, categories, and codes of 6-week data. Information italicized and underlined represents themes or codes unique to 6-week data.

**Figure 2:**
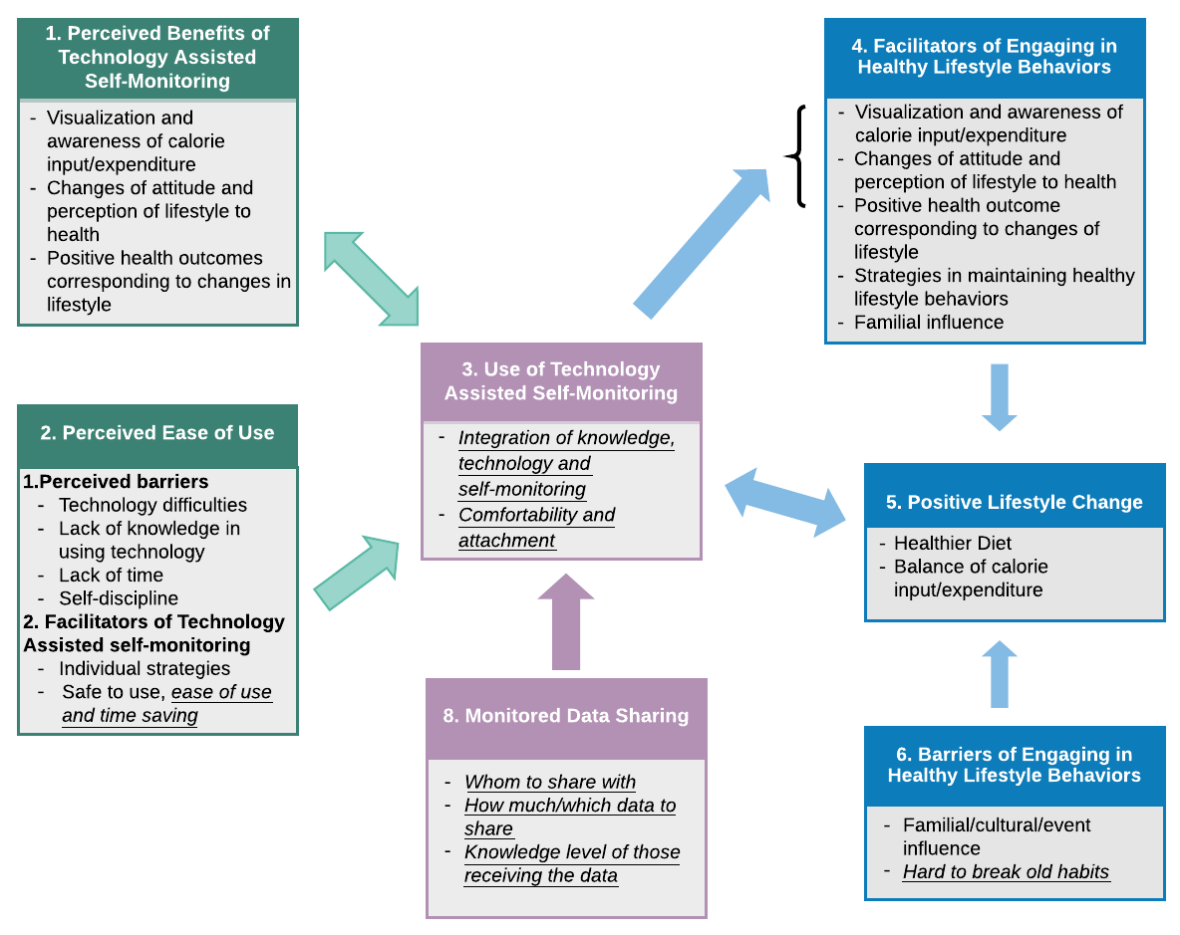
Themes, categories, and codes of 6-month data. Information italicized and underlined represents themes or codes unique to 6-month data.

The following eight major themes emerged from the interview data (Table 2): (1) *perceived benefits of technology-assisted self-monitoring*; (2) *perceived ease of use*; (3) *use of technology-assisted self-monitoring*; (4) *facilitators of engaging in healthy lifestyle behaviors*; (5) *positive lifestyle change*; (6) *barriers of engaging in healthy lifestyle behaviors*; (7) *learning curve*; and (8) *monitored data sharing*. The first six of the eight themes were shared between the 6-week and 6-month timepoints, but the codes within these themes were not all the same and differed slightly between the two timepoints. These differences provide insights into the evolution of participant perceptions of using technology for self-monitoring of lifestyle behaviors and health indicators, as well as the attitudes and changes in lifestyle behaviors, difficulties, and processes through the study. This helped reflect the journeys and adaptations of participants throughout the study by analysis of thoughts and perceptions at each of the respective 6-week and 6-month timepoint discussions. Figure 1 and Figure 2 illustrate the themes and codes for the 6-week and 6-month discussions, respectively.

**Table 2:** Eight themes that emerged from the data.

Theme	Brief description
1. Perceived benefits of technology-assisted self-monitoring	Encompassed the usefulness, helpfulness, and enjoyment of the technology-assisted self-monitoring intervention.
2. Perceived ease of use	Encompassed the perceptions on how difficult, easy, or comfortable the study technology-assisted self-monitoring tools are to use, including barriers and facilitators.
3. Use of technology-assisted self-monitoring	Included the ways in which participants used technology assisted self-monitoring tools that would have an impact on their behavioral health and lifestyle.
4. Facilitators of engaging in healthy lifestyle behaviors	Incorporated the changes in attitude and perceptions of lifestyle to health, awareness, strategies, and other factors regarding how participants impacted their own healthy lifestyle behaviors, as well as how it further influenced their decisions and choices.
5. Positive lifestyle change	Detailed the positive lifestyle changes that have come about from participating in the technology-assisted lifestyle intervention
6. Barriers of engaging in healthy lifestyle behaviors	Encompassed participant comments on times when they came across struggles or barriers to engaging in a healthy lifestyle
7. Learning curve	Encompassed experiences of the learning process and adjustments that took place while participating in the study and learning during the study.
8. Monitored data sharing	Encompassed opinions about with whom to share data and what data to share.

### Shared Themes Between the 6-Week and 6-Month Discussions

There were six themes consistent and shared between the 6-week and 6-month timepoint discussions. These themes encompass the thoughts and reactions that participants shared on their perceptions of the intervention and technology-assisted self-monitoring, and how these perceptions affected self-monitoring behaviors, healthy lifestyle change, and daily life.

#### Theme One: Perceived Benefits of Technology-Assisted Self-Monitoring

This theme encompassed the usefulness, helpfulness, and enjoyment of the technology-assisted self-monitoring intervention. Participants started noticing and deeming benefits right away, which continued through the study, as comments reflecting benefits were found at both timepoints. The perceived benefits from technology-assisted self-monitoring included the direct benefits participants found from technology, such as being able to visually see calorie counts and being more aware of calorie intake versus exercise expenditure.

…See and the phone when you put the food in, what you eat, it always give you like the net amount and it’s kind of like watch out, you only have this much. If you want to eat more, you have to do more exercise.

Speaker F, 6-week discussion

I like it [LoseIt! App]. You get to see visually what you’re eating, how many calories involved. Once you visually see you put the pressure on your brain and you’re remembering next time. It liked it, in spite of whatever you think that you don’t like it. But it was good. I liked it.

Speaker J, 6-month discussion

#### Theme Two: Perceived Ease of Use

This theme encompassed the perceptions on how difficult, easy, or comfortable the technology-assisted self-monitoring tools are to use. This theme had two categories. The first of the two was perceived barriers to technology use, which included participant struggles in the use of technology, such as technical difficulties (eg, logging food), lack of time, and lack of self-discipline.

I still have problems using the phone and putting in my diet. I guess I should do like (speaker A) says and put in your own food instead of searching for something close to it that is, you know, close to what I’m eating.

Speaker C, 6-week discussion

…it’s not that it [recording weight] was hard, it’s that I think I just didn’t do it; not that it was hard. I just didn’t follow through in doing it.

Speaker K, 6-month discussion

The second category was facilitators of technology-assisted self-monitoring, which mainly included the individual strategies and external support (eg, family) that participants employed while using technology to facilitate its use, as well as the perceived safety (comfortability) of the technology in terms of storing and entering health data around others. Under this category, some codes were unique to either the 6-week or 6-month data, which are described in the *Shared Themes With Unique Codes in the 6-Week or 6-Month Discussion* section.

I guess that’s what I should do is carry mine with me all the time. Right now, when I’m out, I write down what I’ve had but I wait until I’m back home to do it. It makes sense if you carry it with you all the time then you can automatically put it in. So that’s what I should do.

Speaker D, 6-week discussion

We try, my wife and I working together and we’re trying to do it (recording food using LoseIt!) as we eat on a daily basis. Whenever we do a meal, we finish a meal, then we put it on. We’ve been working together on it (self-monitoring) slowly, but she fall out on it sometimes… laughter…

Speaker A, 6-week discussion

Don’t nobody know who it is. Even if they’re looking at it, they can’t figure out which person it is. I think it’s pretty much safe.

Speaker K, 6-month discussion

Under *theme two,* there were a few code differences between the 6-week and 6-month focus group interviews. For the facilitation of technology-assisted self-monitoring in 6-week discussions, participants referred to using help from family, friends, and those in the program to learn, work, and understand the technology.

But I had my granddaughter to kind of help me a bit now so I think I’m getting to know how to do it now because she put in a lot of stuff when she showed me how. So I’m getting the hang of it. But I was having a lot of problems putting in stuff. And then there’s a little microphone on there. Like my granddaughter, well then she’s just say what she wanted, so yeah she just said like “baked chicken”, and then on the thing it brings up. I didn’t know that.

Speaker C, 6-week discussion

During the 6-month interviews, however, participant comments reflected a greater knowledge in terms of using technology, in addition to discussing the technology in terms of personal independent facilitation, time-saving features, and safety of the data entered.

It was hard but you know, we did it. We coped with it. We got through it. Had problems with our machines and stuff but we did that…

Speaker C, 6-month discussion

I like the connection. One less step you have to do.

one of the speakers, 6-month discussion

#### Theme Three: Use of Technology-Assisted Self-Monitoring

This theme included the ways in which participants used technology-assisted self-monitoring tools that would have an impact on their behavioral health and lifestyle. Although this theme was shared between the two timepoints, the codes they contained were vastly different and portrayed how participants adapted and learned over time.

Starting at the 6-week discussion, participant comments were focused on the frequency of technology-assisted self-monitoring use, and how their commitment to applying technology-assisted self-monitoring and health education increased during the study.

I’ve been really good about that [monitoring blood glucose]. I put that in as soon as I do it. As soon as I do it, I put the phone right by it and it goes in.

Speaker C, 6-week discussion

At 6 months, discussions on the use of technology-assisted self-monitoring reflected participant integration of knowledge and technology-assisted self-monitoring, and perpetuated being aware of how this can help them in their life. Participants also commented on having greater comfort with the use of technology-assisted self-monitoring, not wanting to give it up at the end of the study, and being able to utilize and integrate study education into behavioral lifestyle.

…I got so now I depends on it, so when you take it back, I’m gonna miss it!

Speaker P, 6-month discussion

By the different information that I received. A lot of the information that I didn’t know, now that I know it. I can take that and use it to the best of my ability, that would help me, in what I need to do daily, you know, as far as eating, exercising. So I like it.

Speaker P, 6-month discussion

#### Theme Four: Facilitators of Engaging in Healthy Lifestyle Behaviors

This theme incorporated the changes in attitude and perceptions of lifestyle to health, awareness, strategies, and other factors regarding how participants impacted their own healthy lifestyle behavior, as well as how it further influenced their decisions and choices. Some of these facilitators (eg, positive health outcomes corresponding to changes in lifestyle) were also benefits participants perceived from using technology-assisted self-monitoring (seen in *theme one*).

…I have my son and I go out try to keep up steps with him. Sunday I got up to 11,000 steps.

Speaker F, 6-week discussion

…it made me aware of the food that I was eating, and my calories intake, and noticing, paying attentions to like what I was eating that was causing my sugar to spike, and I liked it. I really did. Because it was interesting to me, because I wasn’t aware of what I was eating, what I wasn’t eating, when I was eating, so it helped me.

Speaker P, 6-month discussion

Under *theme four*, there were code differences between the 6-week and 6-month discussions. For the facilitators of engaging in healthy lifestyle behaviors at 6 weeks, the code *physical manifestations* *associated with lifestyle change* emerged. This code includes participants discussing how their mind and body reacted differently to food after starting the program, such as salivating and becoming sick or nauseous when reverting back to an old diet.

I come from a family that loves sweets… But once I’ve learned how not to eat and learned, like my daughter bought ice cream, Bluebell the other night, and I took 2 tablespoons and I was going to have a little taste. Well I ate one portion of the half of the first tablespoon, I didn’t want anymore. And I’m a sweet lover, you know I came from that background. But I find that I don’t want it. My body does not want it.

Speaker G, 6-week discussion

…It’s like when you went out over there. When you haven’t eaten greasy foods you start eating… Right, it makes you sick…It makes me nauseated now. I’m telling you, when I smell grease…

Speaker F, 6-week discussion

#### Theme Five: Positive Lifestyle Change

This theme detailed the positive lifestyle changes that have come about from participating in the technology-assisted lifestyle intervention, such as having a healthier diet and being better able to engage in balancing calorie intake versus exercise expenditure.

…it made me aware of the food that I was eating, and my calories intake, and noticing, paying attentions to like what I was eating that was causing my sugar to spike, and I liked it. I really did. Because it was interesting to me, because I wasn’t aware of what I was eating, what I wasn’t eating, when I was eating, so it helped me.

Speaker P, 6-month discussion

#### Theme Six: Barriers of Engaging in Healthy Lifestyle Behaviors

This theme encompassed participant comments on times when they came across struggles or barriers to engaging in a healthy lifestyle, such as family or cultural influences, as well as special or celebratory events that affected food and diet choices.

Yeah [events in your life] that’s kind of hard. Like last night, I’ve got to admit I kind of goofed up last night. My niece had a little birthday party at Marco’s last night, it’s a Mexican restaurant. And I did eat the enchiladas that I probably shouldn’t have.

Speaker C, 6-week discussion

Under *theme six*, there was one code difference between the 6-week and 6-month discussions. Discussions about how to break old habits or having a hard time doing so appeared in the 6-month data but not in the 6-week data.

I’m a night person, so I eat later instead of earlier. I haven’t broke that habit yet. I still eat 7, 8 o’clock. Nine. Just habit.

Speaker J, 6-month discussion

### Themes and Encompassed Codes Unique to Either the 6-Week or 6-Month Discussion

#### Theme Seven: Learning Curve

This theme encompassed codes that were unique to the 6-week discussion. It describes the learning process and adjustments that took place while participating in the study and learning during the study. Many participants referred to the learning curve as a slow process, but one that they were able to “get the hang of” and were willing to complete. The learning curve was fueled by participants using individual learning strategies, help from family and friends, and overall slow but steady adjustments to technology-assisted self-monitoring, program requirements, and behavioral modifications.

When we first started, you kind of, even though it was explained, it was explained in details. But still again, I don’t care how you explained it, the first time you never get it right. So, it’s a slow process and doing it, I’m slowly learning how to register and put the weight in, and also put the sugar in before the, you know before the phones and the meter together. But you know I had an issue with the scale; it wasn’t working right and so we had to reset it again. So these are just some things you want to make a point to, but it’s a slow process, and I’m learning it pretty well and I’m having no problems.

Speaker A, 6-week discussion

#### Theme Eight: Monitored Data Sharing

This theme encompassed codes that were unique to the 6-month discussion. The theme at 6 months showed that participants used several digital self-monitoring tools, which gathered their health data while partaking in the study. Participants expressed who they wanted to share this data with and how much of the data or what data they wanted to share, and expressed the need to ensure that those on the receiving end of the health data are educated on what it means, how to read it, and what its implications are.

Yeah, but on the other hand, if they’re not educated on what’s what, they wouldn’t understand. They’d almost have to have to go to a short study to know what is the reading, what is this, what is that. Cause they wouldn’t know. Like, my daughter, I have to tell her, you see this, you see that.

Speaker J, 6-month discussion

I would like that [to share with diabetes educator].

Speaker K, 6-month discussion

Family. It’s really good detail. And it really helps to share with the family especially, for them to be aware of.

Speaker N, 6-month discussion

## Discussion

### Principal Findings

This study used data collected from focus group discussions at two timepoints (6 weeks and 6 months) after initiation of a lifestyle intervention using technology-assisted self-monitoring of lifestyle behaviors and diabetic health indicators. Despite barriers and challenges encountered during the technology-assisted self-monitoring intervention, overall, participants could identify various resources to overcome barriers, and it was concluded that technology-assisted self-monitoring was beneficial, safe, and feasible to use for positive lifestyle changes. In addition, although the similarities of the findings between the two timepoints were very important and numerous, the differences between them highlighted the progression, adjustment, learning curve, application, and individual strategies associated with the use of the technology, self-monitoring, and lifestyle modifications. Implications for future studies and clinical practice are further discussed below.

This study found that at both 6 weeks and 6 months, technology-assisted self-monitoring facilitated participants’ ability to visualize and learn how their blood glucose reacted to their lifestyle, creating awareness for healthy lifestyle choices to manage diabetes and allowing engagement in healthy lifestyle behaviors. This finding echoes the conclusion of a meta-synthesis study, which concluded that being able to make sense of diabetic factors is critical in diabetes management [37]. Particularly, after reviewing 50 qualitative studies of diabetes self-management, the same study reported that individuals with diabetes frequently experience multiple gaps in their understanding to select appropriate actions and must make sense of new situations in order to construct their new reality. Our findings suggest that technology-assisted self-monitoring of lifestyle behaviors and diabetes-related health indicators helped the study participants understand the importance and rationale of selecting healthy choices and behaviors, and helped to make sense of why certain lifestyles must be adopted to control blood glucose. Health providers, such as diabetes educators, can incorporate this into clinical practice and encourage patients to adopt self-monitoring of their lifestyles and health indicators for better diabetes management.

In turn, visualization of calorie intake and expenditure, as well as how health outcomes correspond with lifestyle changes motivated participants to commit to self-monitoring. However, previous studies have reported that frustration related to high or low glucose readings is one of the barriers of committing to self-monitoring [38]. Therefore, health education may be needed for not only managing glucose control using self-monitoring technologies, but also managing emotions related to glucose fluctuations.

Despite the barriers and challenges participants encountered at the beginning of the intervention, they were able to identify strategies from various resources to overcome obstacles and cope with them. The identified barriers (eg, technology difficulties and lack of time) are similar to those reported previously [39]. Our study found that the involvement of family and friends, as well as the assistance from an intervention program could help overcome barriers and facilitate technology-assisted self-monitoring. Future interventions could consider involving a family member or a friend in the intervention program. Additionally, given the variations in how individuals integrated the process of self-monitoring, future lifestyle interventions may consider individualizing self-monitoring strategies to improve adherence.

Different findings at 6-week and 6-month timepoint discussions were notable. This study identified an overall learning curve in technology-assisted self-monitoring from the 6-week timepoint of the intervention to the 6-month timepoint. The learning curve experienced by participants might have led to the more positive outcomes seen at the 6-month discussion. While working through the learning curve seen at 6 weeks, participants focused more on factors that would help them adapt to technology-assisted self-monitoring, such as getting help from family and friends. At the 6-month discussion, they appeared to be individually sufficient with regard to knowledge and technology in a more experienced way than before. In addition, at 6 months, the identified additional facilitators of engaging in self-monitoring included ease of use and time saving, which were not identified at 6 weeks. A study examining digital health systems for personalized lung disease management reported that patients become faster at completing their digital symptom log over time, which partially confirmed our findings [40]. Further, at the end of the 6-month discussion, participants seemed more comfortable and integrated in using technology-assisted self-monitoring of lifestyle behaviors and health indicators. The barriers of engaging in technology-assisted self-monitoring (eg, technology difficulties) have been extensively studied [34,38,39]. The learning curve of using technology-assisted self-monitoring for disease management, however, has rarely been comprehensively studied. Our study provides findings of initial exploration of the learning curve in technology-assisted self-monitoring. Future studies with longer follow-up are warranted to explore the learning curve for different populations, as well as to determine whether participants would get fatigued with self-monitoring and begin to engage less in self-monitoring overtime, and consequently, relapse back to their previous unhealthy lifestyles.

Lastly, at the 6-month discussion, participants perceived that recording lifestyle data was safe and commented that they were willing to share the recorded data with health care providers, friends, and family members. The relevant questions on sharing health data were not asked at the 6-week interview, so it did not appear in the 6-week discussion. The results are consistent with the findings of previous studies that older adults would like to share their tracked health data with health care providers, friends, and family members [41]. Their willingness to share health data with health care providers may help the physician-patient dyad to better improve patient health outcomes. Additionally, willingness to share data with friends and family members may lead to patients getting help from others for overcoming barriers to engaging in self-monitoring and may encourage positive lifestyles, as identified in this study. Further, awareness among friends and family members about how health indicators correspond to lifestyle behaviors may foster or create a positive atmosphere around patients to promote positive lifestyle changes and better health outcomes. Future lifestyle intervention programs may consider including both patients and their loved ones in diabetes management programs if possible.

### Limitations

There are several limitations in this study. First, all devices and supplies were provided to participants, so the study was not able to identify other important barriers for self-monitoring engagement, such as the costs of health devices, test strips, and lancets [38]. Second, the study used a convenient sample with a small sample size and with all study participants enrolled in a lifestyle intervention program with diabetes management health education provided. The study findings may not be generalizable to other individuals for technology-assisted self-monitoring of lifestyle without health education support. However, our findings highlighted that there is a learning curve when using technology-assisted lifestyle monitoring, and individuals not participating in a health education program may identify various resources to promote self-monitoring and positive lifestyle changes. Third, the study was not designed to explore participants’ perceptions of factors related to positive lifestyle changes. Therefore, the captured factors associated with lifestyle changes were limited in this study.

### Conclusion

Although there were some barriers, the study participants were able to identify various individual and external strategies to adjust to and engage in technology-assisted self-monitoring, and it was concluded that technology-assisted self-monitoring was beneficial, safe, and feasible to use. The learning curve, along with other differences identified between the 6-week and 6-month discussions, suggested the adaptability process of engaging in technology-assisted self-monitoring for diabetes management. These patient perspectives need to be considered in future research studies when investigating the effectiveness of using technology-assisted self-monitoring for diabetes management, as well as in clinical practice when recommending technology-assisted self-monitoring of lifestyle behaviors and health indicators to improve health outcomes.

## Abbreviations

HbA1c: glycated hemoglobin
TAM: technology acceptance model
